# Single-Sample Face Recognition Based on Intra-Class Differences in a Variation Model

**DOI:** 10.3390/s150101071

**Published:** 2015-01-08

**Authors:** Jun Cai, Jing Chen, Xing Liang

**Affiliations:** School of Optoelectronics, Beijing Institute of Technology, Beijing 100081, China; E-Mails: purepurple@bit.edu.cn (J.C.); liangxing1989@126.com (X.L.)

**Keywords:** intra-class variation model differences, face recognition, sparse representation

## Abstract

In this paper, a novel random facial variation modeling system for sparse representation face recognition is presented. Although recently Sparse Representation-Based Classification (SRC) has represented a breakthrough in the field of face recognition due to its good performance and robustness, there is the critical problem that SRC needs sufficiently large training samples to achieve good performance. To address these issues, we challenge the single-sample face recognition problem with intra-class differences of variation in a facial image model based on random projection and sparse representation. In this paper, we present a developed facial variation modeling systems composed only of various facial variations. We further propose a novel facial random noise dictionary learning method that is invariant to different faces. The experiment results on the AR, Yale B, Extended Yale B, MIT and FEI databases validate that our method leads to substantial improvements, particularly in single-sample face recognition problems.

## 1. Introduction

Face recognition has dramatically drawn wide attention due to the advancement of computer vision and pattern recognition technologies [1–3]. Although face recognition systems have reached a certain level of maturity under certain conditions, the performance of face recognition algorithms are still easily affected by external and internal variations, such as background illumination, pose, facial expressions, aging and disguises. Many well-known algorithms have been proposed to overcome these challenging problems [4–10]. Appearance-based methods have been widely used in face recognition. The common goal of these methods is to learn a compact feature subspace for face recognition in a supervised, semisupervised, or unsupervised manner, in such a way that the intrinsic characteristics of the original face samples are well preserved [11]. Representative algorithms include the Eigenfaces [12,13], Fisherfaces [14], aplacianfaces [15], marginal fisher analysis [14], and their weighted, kernelized and tensorized variants [16–20]. More recently, sparse representation-based classification (SRC) has shown inspiring results in the face recognition field [21]. Subsequently, there has been an increasing interest in SRC and plenty of studies have sought to improve its performance. Low-rank matrix recovery was introduced in the SRC framework. The structural incoherence separates the frontal face from occlusions and disguises presented both in the training and testing face images [22–26]. In addition, a dictionary learning framework that combines the block/group or reconstructed block/group sparse coding schemes is also proposed [27].

Although very promising results have been achieved in the works mentioned above, their requirement for acquiring a large amount of training images might not be practical. In fact for mass surveillance tasks (e.g., law enforcement, e-passports, driver licenses, *etc.*), high recognition performance under the condition of only a single training face image per person is required, because it is normally difficult to collect additional samples under these scenarios [28]. The performance of the appearance-based face recognition methods, however, is heavily influenced by the number of training samples per person [29]. More specifically, if the number of training sample per person is much smaller than the feature dimension of face samples, it is generally inaccurate to estimate the intra-personal and inter-personal variations. For instance, LDA will degenerate to PCA [30]. This is the so-called Single Sample Per Person (SSPP) problem in face recognition [31]. Therefore, many existing appearance-based methods cannot be directly applied for SSPP due to the imprecise between-class and within-class scatter.

As cited in [32,33], SRC needs adequate training images of each subject that contain different facial variations of that subject to ensure success of the sparsity-based recognition method. For a single sample face recognition problem, this approach will perform poorly. To address this issue, in [34], the authors introduced a sparse illumination transfer technique to compensate the missing illumination information typically provided by multiple training images. Deng *et al.*, applied an auxiliary intra-class variant dictionary to represent the possible variation between the training and testing images [35]. Furthermore, they challenge the SSPP problem by proposing a “prototype plus variation” representation dictionary which is assembled by the class centroids and the sample-to-centroid differences for sparsity-based face recognition [36].

The above algorithms can be summarized as a generic training set is adopted to extract the discriminatory information which should be adapted to identify other persons. Unfortunately, these algorithms would fail in constructing the discriminatory dictionary that represents intra-personal variations of different persons and the inter-personal variations for different persons.

Inspired by the observation and prior work on sparse representation single sample face recognition [35,36], we present an intra-class facial variation modeling system for sparsity-based SSPP problem. In our model, the facial sample is regarded as a sparse linear combination of the class centroid and the intra-class difference of the variation model, which will lead to an enormous improvement under uncontrolled training conditions.

The main contributions of the research reported in this paper can be summarized is the following three aspects: first we analyze the structural incoherence of the derived facial variation basis, that is, the intra-class similarity and intra-class difference of the facial variation are introduced for modeling various facial differences more accurately; second, our proposed various facial variation models can be constructed from subjects outside the training samples; third, the whole face recognition process takes place in the compressive sampling domain, which is 16 times faster than image-based face recognition algorithms.

The remaining of this paper is organized as follows: Section 2 reviews related works on SRC for face recognition and facial variation modeling systems for SSPP. In Section 3, we present our face recognition algorithm based on modeling various facial variation. Experimental results on the AR, Yale B, Extended Yale B, MIT and FEI databases are presented in Section 4. Finally, Section 5 concludes this paper.

## 2. Background of Our Algorithm

In this section, we first briefly introduce some typical face recognition systems which are the foundation of our modeling system.

### 2.1. Sparse Representation Based Face Recognition

Since our classification algorithm is based on SRC, we now briefly review this algorithm for the sake of clarity. The SRC-based face recognition algorithm considers each test image as a sparse linear combination of training image data by solving a *ℓ*_1_ minimization problem. Assume a face image in grayscale can be written in vector form by stacking its pixels. In the training stage, given *k* training subject classes, denote *n* well-aligned training images as the matrix ***D***=[***D***_1_,***D***_2_,…,***D****_k_*] ∈ ℝ*^d^*^×^*^n^*, where the sub-matrix ***D****_i_* ∈ ℝ*^d^*^×^*^n_i_^* (*i*=1,…,*k*; $n=\sum_{i=1}^{k}n_{i}$) of the same dimension as *x* are sampled for the *i*-th class under the frontal position and various facial variation conditions. Then the linear representation of a testing sample *x* can be rewritten as:

(1) x=Dα+z

If *x* belongs to the *i*-th class, then *x* lies in the low-dimensional subspace spanned by the training images in ***D****_i_* Namely, the coefficient matrix *α* =[0,…,0…, *α_i_*_1_,… *α_in_i__*,…,0,…,0]^T^ ∈ ℝ*^n^* is a vector whose entries are zeros expect those associated with *i*-th class. *z* is a noise term with bounded energy ‖*z*‖_2_ < *ε*. The theory of compressive sensing reveals that if the solution of *α* is sparse enough, it can be recovered efficiently by the following *ℓ*_1_ minimization problem:

(2) $$\min{\left\|\alpha \right\|}_{1}s.t.\text{x}=\text{D}\alpha +z$$

### 2.2. Single Sample Face Recognition with the Facial Variation Dictionary

The previous studies in [22,32,33] have revealed the limitations of sparsity-based recognition when the training images are corrupted and the number of samples per class is insufficient. In [35], the authors utilize the intra-class variant bases to represent unbalanced lighting changes, exaggerated expressions or occlusions that cannot be modeled by the small dense noises *Z*. Based on this perspective, they further proposed a prototype plus variation (P + V) model and a corresponding sparsity based classification algorithm which they called superposed SRC (SSRC). The prototype plus variation model assumes that the observed signal is a superposition of two different sub-signals *x_p_*, *x_v_* and noise term *z* (*i.e.*, *x*=*x_p_* +*x_v_* +*z*). *x_p_* is sparsely generated with a prototype dictionary ***P***=[*P*_1_, *P*_2_,…,*P_k_*] ∈ ℝ*^d^*^×^*^k^*, where the sub-matrix *P_i_* ∈ ℝ^d×1^ stacks the prototypical base of class *i*. Similarly, *x_v_* is sparsely generated with a variation dictionary ***V*** ∈ ℝ*^d^*^×^*^n^* represents the universal intra-class variant bases. Then, the linear representation of a testing sample *x* can be written as:

(3) $$x=\text{P}\alpha_{0}+\text{V}\beta_{0}+z$$

where the prototype dictionary ***P*** ∈ ℝ*^d^*^×^*^k^* can be represented as follows:

(4) $$\text{P}=[c_{1},\ldots ,c_{i},\ldots ,c_{k}]\in {\mathbb{R}}^{d\times k}$$

where $c_{i}=\frac{1}{n_{i}}{\text{D}}_{i}e_{i}$ is the geometric centroid of each class and *e_i_* =[1,…,1]*^T^* ∈ ℝ*^n_i_^*^×1^. The variation matrix ***V*** ∈ ℝ*^d^*^×^*^n^* is naturally constructed by the sample based difference to the centroids as follows:

(5) $$\text{V}=[{\text{D}}_{1}-c_{1}e_{1}^{\text{T}},\ldots ,{\text{D}}_{k}-c_{k}e_{k}^{\text{T}}]\in {\mathbb{R}}^{d\times n}$$

Hence the sparse representation *α*_0_ and *β*_0_ can be solved by using *ℓ*_1_ minimization method.

## 3. Compressive Single Sample Face Recognition with Improved Facial Variation Dictionary

### 3.1. Improved Facial Variation Dictionary Learning

For real-world face recognition problems, when the number of samples per class is insufficient, particularly when only a single sample per class is available, the SRC-based framework would collapse. However, we cannot expect that the training image data can be always collected in well-controlled settings. Besides illumination, pose, and expression variations, it is possible that one can be wearing a scarf, gauze mask, or sunglasses when the face image is taken by the camera. As discussed in Section 2.2, facial variation modeling system can be applied to alleviate the aforementioned problem by decomposing the collected data matrix into two different parts. One is a representative basis matrix of the prototype for each class and another is the associated facial variation caused by variable expressions, illumination and disguises, which can be shared across different subjects.

**Figure 1.**
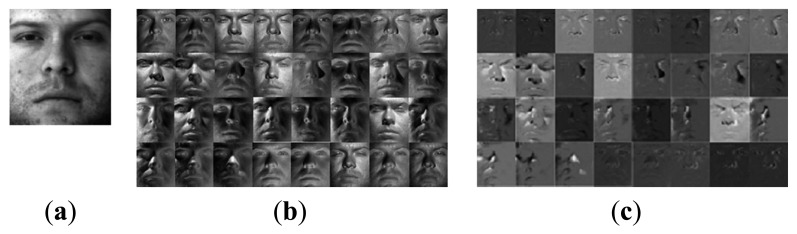
The illustrative examples of the facial variation model based on Yale B database. (**a**) The “prototypes” derived by averaging the images of the same subject; (**b**) The “sample-to-centroid” variation images of SSRC method; (**c**) The intra-class difference of the “sample-to-centroid” variation images of our method.

Actually, a SSRC algorithm constructs an intra-class variant dictionary to represent the possible variation between the training and testing images. However, the proposed intra-class variant dictionary contains not only the possible variation information, but also the associated facial information (see Figure 1). From Figure 1 we can see clearly that the (P + V) model-based facial variation dictionary introduced in SSRC algorithm comprises the specific subject (see Figure 1b), and our proposed model-based facial variation dictionary basically consists of various facial variations (see Figure 1c), and the reference subjects outside the training subjects also can provide the facial variant bases since the variations of different subjects are sharable. In [35], the authors also mentioned that the intra-class facial variation of different subjects is similar since the shapes of human faces are highly correlated, and these similarly shaped faces can be readily found if the data set contains a sufficiently large number of subjects.

Figure 1 illustrates a typical example of the difference between SSRC and our method. Both Figure 1b,c are the facial variation bases derived from this specific class via SSRC and our method separately. From Figure 1 we can see that our method can provide additional discriminating ability to the facial variation dictionary ***V*** by promoting its structural property.

**Figure 2.**
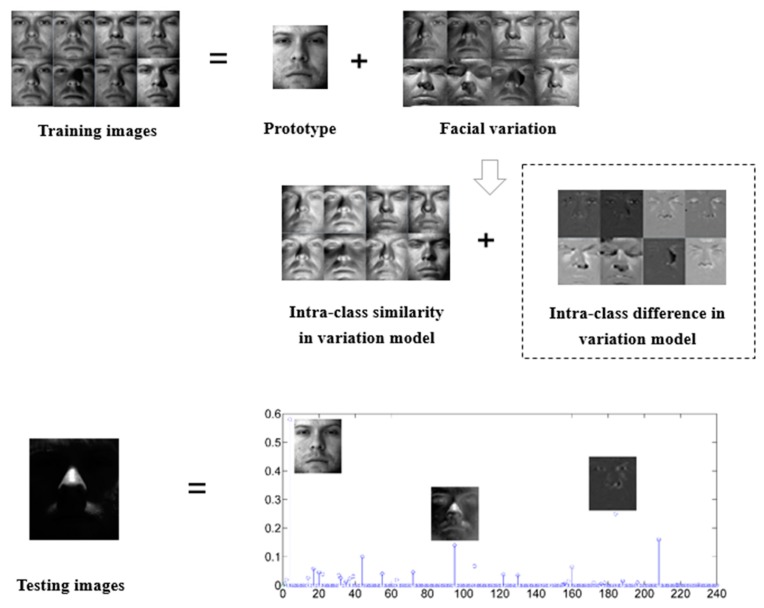
The basic idea of our method. (**Top**) The intra-class variant basis derived from the reference person can be shared by other people; (**Bottom**) The nonzero coefficients of the sparse representation are expected to concentrate on the training samples with the same identity as the test sample and on the related intra-class similarity and difference of facial variant bases.

Inspired by [36] and the observation of structural property of derived facial variation basis, we propose to promote the incoherence between the facial variation matrices. Figure 2 illustrates this simple idea of our method to address the challenging SSPP problem. For a specific subject, we further decompose facial variations dictionary ***V*** into a low rank intra-class similarity ***E*** and associated sparse intra-class difference ***G*** respectively.

As illustrated in Figure 2, in our method a testing sample *x* is represented as a sparse linear combination of the class centroid ***P***, the intra-class similarity of variation ***E*** and the intra-class difference of variation ***G***, which can be written as:

(6) $$x=\text{P}\alpha_{0}+\text{V}\beta_{0}+z=\text{P}\alpha_{0}+\text{E}\eta_{0}+\text{G}\gamma_{0}+z$$

Augmented Lagrange multipliers (ALM) [37] are applied to extract intra-class similarity ***E*** and intra-class difference ***G*** from the facial variation dictionary ***V*** as follows:

(7) $$\begin{array}{l} \underset{\text{E},\text{G}}{\min}\text{rank}(\text{E})+\mu {\left\|\text{G}\right\|}_{0}\\ s.t.x=\text{P}\alpha_{0}+\text{E}\eta_{0}+\text{G}\gamma_{0}+z \end{array}$$

To separate the intra-class similarity ***E*** and the intra-class difference ***G***, low-rank minimizes the rank of ‖***E***‖_0_ while reducing to derive ‖***G***‖_0_ the low-rank approximation of ***V***. In Equation (7), we use the nuclear norm ‖***E***‖_*_ (*i.e.*, the sum of the singular values) approximates the rank of ***E***, and the *ℓ*_1_-norm ‖***G***‖_1_ to replace the *ℓ*_0_ -norm‖***G***‖_0_, which sums up the absolute values of entries in ***G***:

(8) $$\begin{array}{l} \underset{\text{E},\text{G}}{\min}{\left\|\text{E}\right\|}_{*}+\mu {\left\|\text{G}\right\|}_{1}\\ s.t.x=\text{P}\alpha_{0}+\text{E}\eta_{0}+\text{G}\gamma_{0}+z \end{array}$$

As seen from Equation (8), the recognition problem is cast as finding a sparse representation of the test image in terms of a superposition of the class centroids and the intra-class difference of facial variant bases. The nonzero coefficients *α*_0_ are expected to concentrate on the same class as the training sample. Therefore the test sample *x* from class *i* can be represented as a sparse linear combination of the corresponding class centroids ***P****_i_*, intra-class similarity ***E*** and the intra-class difference ***G***. If the number of classes *k* is reasonably large, the combination coefficients in *α*_0_ is naturally sparse. If there are redundant and over-complete facial variant bases in ***E*** and ***G***, the combination coefficients in *η*_0_ and *γ*_0_ are naturally sparse. Hence, the sparse coefficients *α*_0_, *η*_0_ and *γ*_0_ can be recovered simultaneously by *ℓ*_1_ -norm minimization.

**Figure 3.**
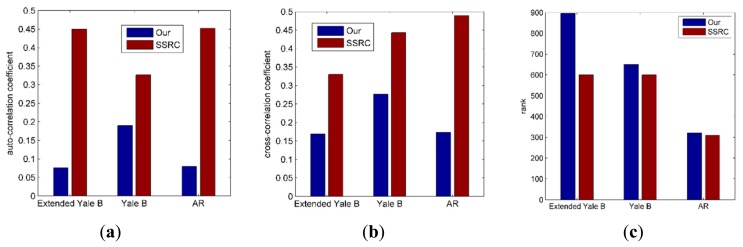
The comparative results of low-rank minimization optimization under difference face datasets. (**a**) auto-correlation coefficient of facial variation bases; (**b**) cross-correlation coefficient between facial variation bases and prototype base; (**c**) the rank of facial variation bases.

In order to prove the low-rank estimation method feasible, we evaluate the auto-correlation coefficient of the same subject, the cross-correlation coefficient of different subjects and the rank of both facial variation ***V*** and the intra-class difference ***G*** under difference face datasets. From Figure 3a we can see that the auto-correlation coefficient of intra-class difference of facial variant bases ***G*** is much lower than and the facial variant bases ***V***. In Figure 3b, the blue bins show the cross-correlation coefficient between intra-class difference of facial variant bases ***G*** and prototype bases ***P***. Similarly, the red bins are the cross-correlation coefficient between facial variant bases ***V*** and prototype bases ***P***. This means our method significantly decreases the correlation of facial variation, that is, the highly related human face is eliminated and ***G*** only represents various facial variation. Figure 3c described that, when the intra class difference is involved, the rank is reduced as we expected. In additional, the results become obvious when the facial variations are diverse.

In this paper, we assume that all test images are well-aligned to simplify our experiments. In fact image alignment and recognition can be achieved robustly within the sparse representation framework mentioned above. Now suppose that the test image *x*_0_ is subject to some misalignment, so instead of observing *x*_0_, we observe the warped image *x* = *x*_0_ o *T*^−1^. Here *T* is a transformation matrix acting on the image domain. If the transformation *T*^−1^ can be found, then we can apply its inverse to the test image and it again becomes possible to find a sparse representation of the resulting image (see Equation (9)). In this case the single-sample alignment approach [38] can be applied in our single-sample face alignment problem:

(9) $$\begin{array}{c} \underset{\text{E},\text{G}}{\min}{\left\|\text{E}\right\|}_{*}+\mu {\left\|\text{G}\right\|}_{1}\\ s.t.x\circ T^{-1}=\text{P}\alpha_{0}+\text{E}\eta_{0}+\text{G}\gamma_{0}+z \end{array}$$

### 3.2. Face Recognition on Compressive Sampling Space

Face recognition requires adequate high resolution samples, so one key issue is how to reduce dimensionality while maintaining the subspace invariance. Recently, compressed sensing (CS) has become one of the standard signal processing methods of computer vision and pattern recognition. Thus, whether CS can be applied to face recognition has been a problem people are keenly concerned about. In [39], the authors suggest that as long as the number of features is large enough, even randomly chosen features are sufficient to recover the sparse representation. More details about applying compressive sensing to SRC are illustrated in [39,40].

Therefore in this paper, CS is introduced to our improved (P + V) model and sparse coefficients are solved in compressed sampling space to further accelerate our face recognition algorithm as:

(10) $$\begin{array}{l} \underset{\text{E},\text{G}}{\min}{\left\|\Phi \text{E}\right\|}_{*}+\mu {\left\|\Phi \text{G}\right\|}_{\text{1}}\\ s.t.y=\Phi x=\Phi (\text{P}\alpha_{0}+\text{E}\eta_{0}+\text{G}\gamma_{0}+z) \end{array}$$

Here the feature dictionary ***A*** =[***P E G***], ***A*** ∈ ℝ*^d^*^×^*^n^* in Equation (10) is substituted by a random projection dictionary ***D*** = ***Φ******A***,***D*** ∈ ℝ*^m^*^×^*^n^* (*m* ≪ *n*) , which can be considered as a compressive measurement of original feature dictionary *A*. Clearly, the dimension of dictionary ***D*** is reduced by using random projection matrix *Φ*. Mathematically *rank*(*AB*) ≤min{*rank*(***A***), *rank*(***B***) }, the rank of ***ΦP*** and ***ΦE*** is smaller than that in Equation (10), which will accelerate the rate of iteration convergence obviously and hence make our algorithm faster. This is also cited in [41], the authors proved the robustness of sparse classifier, group sparse classifier and the nearest neighbor classifier to random projection dimensionality reduction. Algorithm 1 summarizes the details of our recognition algorithm.


**Algorithm 1** Compressive sparse representation-based classification.
**Input:** Training data ***D***=[***D****_1_*, ***D****_2_*, … ***D****_k_*] from *k* classes and the test input y parameter *λ, μ, η*Step 1: Apply Gabor transform on ***D*, *D*** ←*Gabor*(***D***)Step 2: Project random projection onto ***D***, ***D*** ← **Φ** (***D***)Step 3: Extract improved facial variation dictionary from ***D*** **for***i*=1:*N* do
${\text{P}}_{i}=c_{i}=\frac{1}{n_{i}}{\text{D}}_{i}e_{i}$
${\text{V}}_{i}={\text{D}}_{i}-c_{i}e_{i}^{T}$
$\underset{\text{E},\text{G}}{\min}{\left\|{\text{E}}_{i}\right\|}_{*}+\mu {\left\|{\text{G}}_{i}\right\|}_{1}s.t.{\text{V}}_{i}={\text{E}}_{i}+\lambda {\text{G}}_{i}$**end for** Step 4: Perform SRC on ***V***
$\underset{\alpha ,\beta ,\gamma }{\min}{\left\|y-\left[\begin{array}{ccc} \text{P} & \text{E} & \text{G} \end{array}\right]{\left[\begin{array}{ccc} \alpha & \beta & \gamma \end{array}\right]}^{\text{T}}\right\|}_{2}^{2}+\eta {\left\|\begin{array}{ccc} \alpha & \beta & \gamma \end{array}\right\|}_{1}^{\text{T}}$ **for***i*=1:*N* do
$e\left(\text{i}\right)={\left\|y-\left[\begin{array}{ccc} \text{P} & \text{E} & \text{G} \end{array}\right]{\left[\begin{array}{ccc} \alpha & \beta & \gamma \end{array}\right]}^{\text{T}}\right\|}_{2}^{2}$**end for****Output:***y* ← arg min*_i_ e*(*i*)


## 4. Experimental Results

In this section, we will present comprehensive experiments to demonstrate the performance of our recognition algorithm. Our algorithm is evaluated on the following publicly available datasets: AR face database [42], Yale B database [43], Extended Yale B database [44], MIT database [45] and FEI database [46]. Our approach is compared with several other algorithms including the SRC [29] and SSRC [36] under the same conditions whereby all methods are optimized by using the L1-Homotopy [47,48] algorithm with the regularization parameter *λ* =0.005. All experiments are done on a PC with an Intel i7 2600 CPU and 16 G RAM using a single thread. The implementation of all methods is on the Matlab 2013b platform.

**Figure 4.**
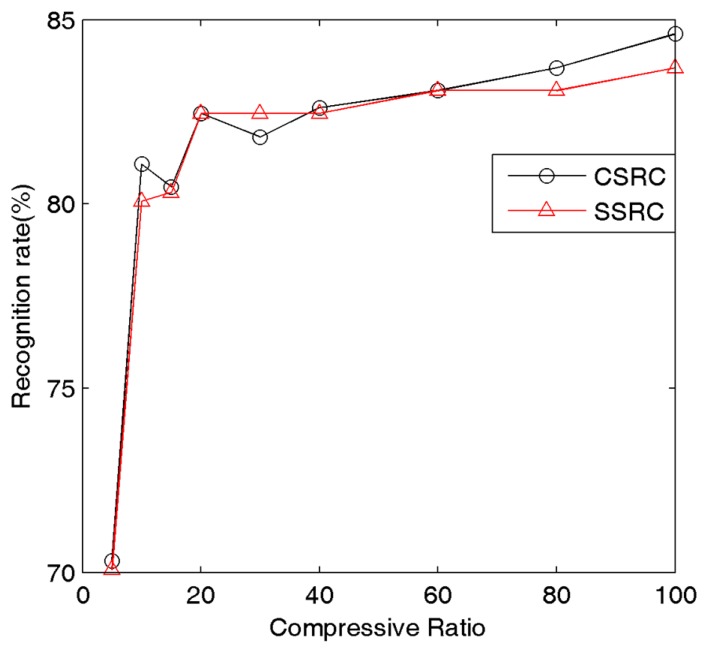
The relationship between the compressive sampling ratio and recognition rate of our method and SSRC for the AR Face Dataset.

To simply test the recognition rate for different compressive ratios we compare the recognition results under different compressive sampling ratios. Figure 4 illustrates the relationship between the compressive sampling ratio and recognition rate of our method under AR Face Dataset. Here 50 subjects from AR face database are chosen, and the images are cropped with dimensions 165 × 120. For each subject, half the images are for training, and the rest for testing. The experimental results give the best sampling ratio which can save memory without sacrificing the recognition rate. From Figure 4, we can clearly figure that 20% of the original dataset is adequate. In Table 1 we record the average elapsed time of SSRC and our method for each test image on the Matlab platform. According to Table 1, with the decrease of sampling rates, our method will be 2–10 times faster than the SSRC method. Therefore, in our following experiments we set the sampling ratio as 20%. For fair comparison, this ratio is applied in our method, SSRC and SRC in the subsequent experiments.

### 4.1. AR Database

**Figure 5.**
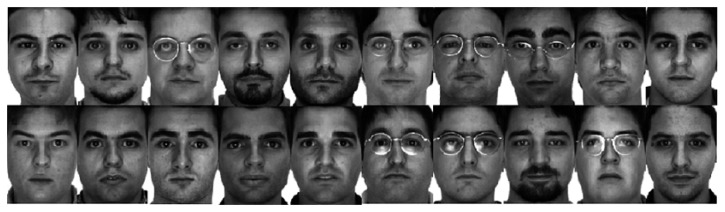
Some specific selected subjects from AR Face Dataset in our experiment.

The AR database consists of over 3000 frontal images of 126 individuals. There are 26 images of each individual, taken at two different occasions. The faces in AR contain variations such as illumination change, expressions and facial disguises (*i.e.*, sun glasses or scarf). We randomly selected 50 subjects for our experiments, and the images are first cropped with dimension 165 × 120, then the 3960D random feature vector is extracted to form the random face. For each subject, 14 images with illumination and expression change, and 12 images with disguise. Figure 5 shows some specific selected subjects.

**Figure 6.**
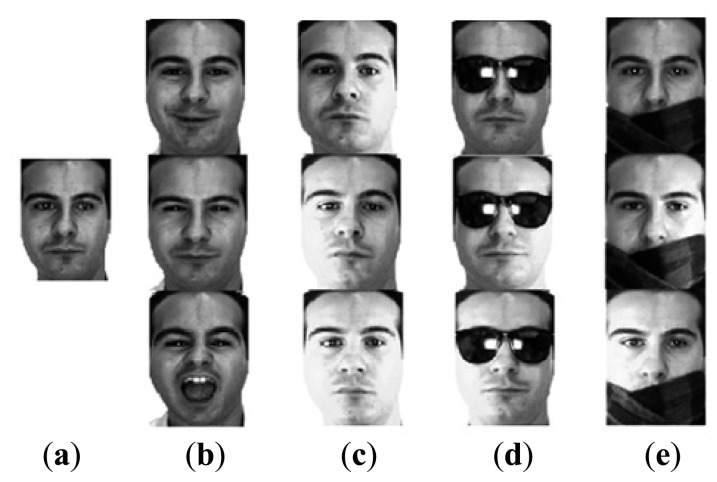
The cropped images of a subject in AR Face Database. (**a**) Neutral expression; (**b**) Expression changes; (**c**) Illumination changes; (**d**) Disguise with sunglasses; (**e**) Disguise with scarf.

The first experiment is executed to test the complex variation effect. For this experiment, images from Session 1 are taken for training, and Session 2 for testing. SSRC obtains a better recognition rate of 81.0769%, which is compared to a 77.0769% recognition rate of SRC, among these algorithms, our method receive 84.6154% recognition rate (see Table 2). Table 2 indicates that once the training sets contain corrupted images (see Figure 6d,e), the occlusion will be regarded as the feature of the subject corresponding to training images. However, the discrimination power of facial variation dictionary is introduced in our method to obtain better presentation.

The second experiment is a reproduction of that in Equation (9) which we evaluate our method by testing the robustness of these algorithms against various intra-class variations based on a single training image per subject. We randomly choose 50 subjects from 126 individuals in the Session 1 of AR database. To construct the intra-class difference of the facial variation, five subjects served as reference subjects. For each subject of the remaining subjects, one single neutral expression image for training, and the other 12 images with four types of variation, *i.e.*, pose, illumination, disguise with sunglasses and scarf for testing (see Figure 6).

As illustrated in Figure 6, the following variabilities are taken into consideration: expression, illumination, disguise, and disguise + illumination. To better understand the effects of each scenario. Table 3 separately enumerates the recognition rates of the four test variabilities. Table 4 enumerates the average recognition rates of this experiment. One can see from Table 4 that the recognition rate increases by switching SSRC to our proposed algorithm.

### 4.2. Yale B and Extended Yale B Database

The Yale B database contains 5760 single light source images of 10 human subjects, each with about nine poses and 64 images taken under various illumination conditions. For every subject in a specific pose, we only use the first subject with 64 aligned frontal images in our experiment. The images are first cropped with dimension 192 × 168, then the 6451D random feature vector is extracted to form the random face. We randomly select three from the 10 people as the reference subjects to construct the facial variation dictionary. For the remaining subjects, we select the neutral face for training, and the remaining for testing.

To further clarify the effect of the illumination, The Extended Yale B database is used. The extended Yale Face Database B contains 16,128 images of 28 human subjects under nine poses and 64 illumination conditions. The data format of this database is the same as the Yale Face Database B. Therefore, like the previous experiment, we randomly select three from the 28 people as the reference subjects to construct the facial variation dictionary. For the remaining subjects, we select the neutral face for training, and the remaining for testing. Table 5 shows the experimental results. For the Yale B and Extended Yale B database, our method achieves very competitive performance since these datasets contain a variety of illumination changes.

**Figure 7.**
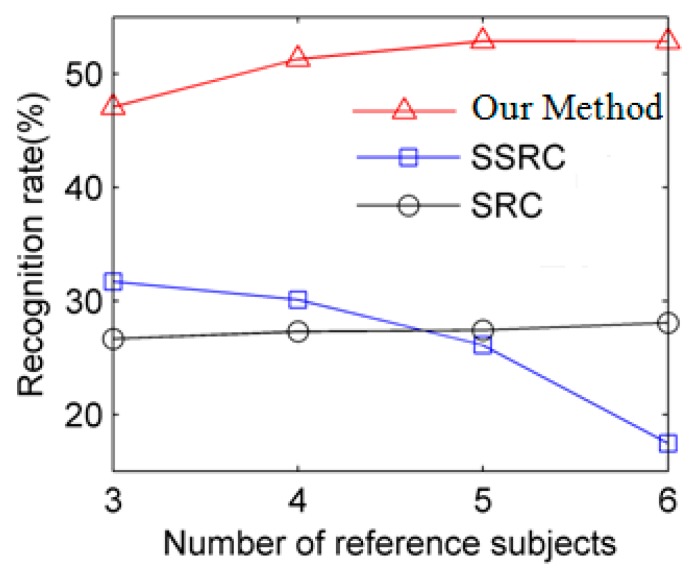
Comparative recognition rates for 3 to 6 reference subjects in the Extended Yale B dataset.

To further carefully compare these algorithms, different numbers of reference subjects from 3 to 6 are presented in Figure 7. As can be seen from Figure 7, we can clearly learn that our method demonstrates its great superiority over SSRC.

### 4.3. MIT Database

The MIT-CBCL face recognition database contains face images of 10 subjects. The test set consists of 200 images per subject. All the training face images are manually cropped into 60 × 60 pixels based on the locations of eyes out-corner points. We randomly select three from the 10 people as the reference subjects to construct the facial variation dictionary. For the remaining subjects, we select the neutral face for training, and the remaining for testing. Table 6 shows that adding intra-class differences to facial variation bases can meaningfully improve their performance by 4%.

### 4.4. FEI Database

**Figure 8.**
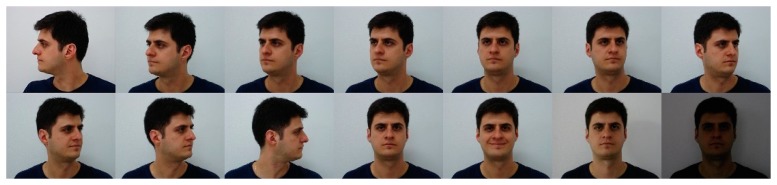
All 14 images of an individual in FEI Face Database.

The FEI face database is a Brazilian face database that contains a total of 2800 images, 14 images for each of 200 individuals. All images are colorful and taken against a white homogenous background in an upright frontal position with a profile rotation of up to about 180 degrees. In our experiment, all samples are cropped into 640 × 480 pixels and converted to gray scale. We randomly select 10 individuals to complete this experiment. To construct the intra-class difference, six subjects (overlapping with the 10 individuals) are selected for training, with 14 images per subject. For the remaining four subjects, the neutral facial image is used for training, the other 13 images are for testing. Figure 8 shows all 14 images of an individual in FEI Face Database.

**Figure 9.**
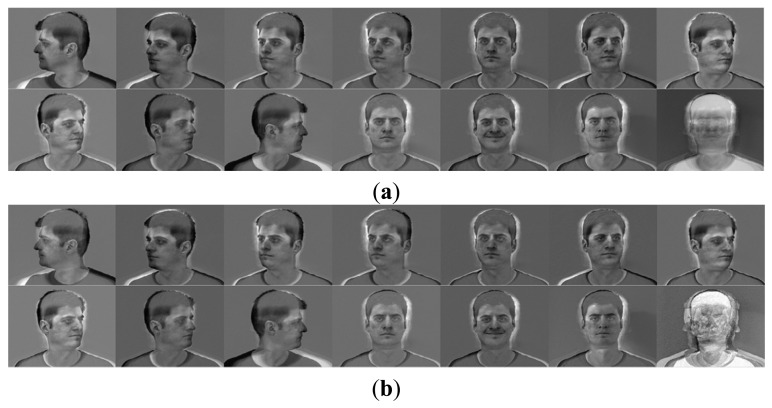
The illustrative examples of the facial variation model based on FEI database. (**a**) The intra-class difference of the “sample-to-centroid” variation images of our method. SSRC method; (**b**) The intra-class difference of the “sample-to-centroid” variation images of our method.

The results shown in Table 7 indicate that adding intra-class differences to facial variation bases can improve the recognition accuracy by 1%. The improvement of recognition performance is not significant compared with experimental results obtained in AR, YALE B and MIT databases. In order to further study this question, we tested the intra-class difference of the “sample-to-centroid” variation images, which we show in Figure 9.

As illustrated in Figure 9, the intra-class difference of the “sample-to-centroid” variation images of SSRC and our method is quite the same, which is significantly different from the results obtained in AR, Yale and MIT databases. A tentative inference on this result is that the “sample-to-centroid” variation images of the same subject are quite different due to the sharp head pose changes. This makes it more difficult to distinguish the prototype (*i.e.*, the frontal facial information) from the intra-class difference of variation.

## 5. Conclusions and Future Work

In this paper, we introduce a low-rank approximation for single sample face recognition. The primary contribution of the proposed method was to help single sample face recognition algorithms to construct facial variation bases for separating the frontal, neutral faces from various facial changes. This method applies congener learning to facial variation modeling and remains robust to light, expression, pose and disguise. We tested the method on several well-known databases. The experiments are conducted under uncontrolled training set and single-sample training set conditions. Our extensive experimental results validate that our method greatly improves the performance of the existing algorithms if the intra-class difference in variation is introduced. Nevertheless, the experimental results in Section 4.4 indicate that our method needs a well-learned dictionary to achieve higher performance. Meanwhile significant head pose changes remain a more challenging problem. We need to work toward the under-sampled open-set face database.

## Figures and Tables

**Table 1.** The running speed for average facial images of SRC and our method on the Matlab platform.

	**SSRC**	***Our* 100%**	***Our* 60%**	***Our* 40%**	***Our* 20%**	***Our* 5%**
AR database	1.48 s	1.51 s	0.75 s	0.58 s	0.32 s	0.17 s

**Table 2.** The comparative recognition rates between SRC, SSRC and our method on the AR data set with different kinds of corrupted training images.

**Algorithm**	**Accuracy**
Our method	84.62
SSRC	81.07
SRC	77.08

**Table 3.** Comparative recognition rates of SRC, SSRC and our method on the AR database using a single training sample per person.

**Methods**	**Expression**	**Illumination**	**Sunglass**	**Scarf**
Our method	70.62	78.64	54.27	50.33
SSRC	68.69	75.31	51.81	46.68
SRC	68.07	34.09	31.57	25.13

**Table 4.** Comparative recognition rates of our method and other recognition methods on the AR Database using a single training sample per person.

**Algorithm**	**Accuracy**
Our Method	61.18
SSRC	55.99
SRC	34.92

**Table 5.** Comparative recognition rates of our method and other recognition methods with three reference subjects in different datasets.

**Algorithm**	**Accuracy**	

	**Yale B**	**Extended Yale B**
Our Method	71.89	47.62
SSRC	63.83	32.79
SRC	40.52	27.66

**Table 6.** Comparative recognition rates for three reference subjects in the MIT database.

**Algorithm**	**Accuracy**
Our Method	70.31
SSRC	66.36

**Table 7.** Comparative recognition rates with 6 reference subjects on FEI database.

**Algorithm**	**Accuracy**
Our Method	61.31
SSRC	60.49
